# Radiolabeling molecular biomarkers of invasive pituitary adenomas: a narrative review

**DOI:** 10.1007/s11102-025-01598-x

**Published:** 2025-11-09

**Authors:** Charbel Marche, Julia C. M. Knop, Shivashankar Khanapur, Eric Suero Molina, Mattew McCord, Vanessa Smith, Michael P. Catalino

**Affiliations:** 1 School of Medicine and Data Science, University of Virginia, Charlottesville, VA USA; 2 Charité – Universitätsmedizin Berlin, corporate member of Freie Universität Berlin and Humboldt-Universität zu Berlin, Berlin, Germany; 3 Radiology and Medical Imaging, University of Virginia, Charlottesville, VA USA; 4 Department of Neurosurgery, University Hospital Münster, Münster, Germany; 5 Department of Pathology, University of Virginia, Charlottesville, VA USA; 6 Departments of Neurosurgery and Medicine, Division of Endocrinology and Metabolism, University of Virginia, Charlottesville, VA USA

**Keywords:** Pituitary adenoma invasiveness, Molecular biomarkers, Radiolabeling, MMPs, VEGF, Survivin

## Abstract

**Purpose:**

Pituitary adenoma, are common intracranial neoplasms that can exhibit invasive behavior, leading to increased morbidity, recurrence, and resistance to treatment. Identifying biomarkers associated with tumor invasiveness could improve early diagnosis and guide therapeutic interventions. This review evaluates molecular biomarkers linked to pituitary adenoma invasiveness and explores the potential of radiolabeling for noninvasive detection.

**Methods:**

A systematic search of PubMed and Embase databases was conducted to identify studies evaluating molecular markers associated with invasive pituitary adenoma. Biomarkers were selected based on their proposed role in tumor invasion, and evidence supporting their clinical relevance was summarized. Additionally, existing radiolabeling techniques for biomarker detection were reviewed.

**Results:**

Five key biomarker groups were identified: matrix metalloproteinases (MMPs), urokinase plasminogen activator (uPA) system, myosin 5 A (MYO5A), vascular endothelial growth factor (VEGF), and survivin. MMPs were strongly linked to extracellular matrix degradation and invasion, while uPA facilitated invasion via MMP activation. MYO5A and survivin were implicated in epithelial-mesenchymal transition and tumor motility, and VEGF promoted angiogenesis. Radiolabeling techniques for MMPs, uPA/uPAR, VEGF, and survivin demonstrated feasibility for imaging tumor invasiveness, though limitations such as non-specific tracer accumulation remain.

**Conclusions:**

This review highlights the potential of molecular biomarkers in predicting pituitary adenoma invasiveness and the emerging role of radiolabeled probes in noninvasive imaging. Future research should focus on validating these biomarkers in longitudinal studies and refining radiolabeling techniques to improve diagnostic accuracy and therapeutic targeting of invasive pituitary adenomas.

## Introduction

Pituitary adenomas are clonal proliferations of specific cell lineages originating in the anterior pituitary gland, as outlined in the WHO Classification of Endocrine and Neuroendocrine Tumors, 5th edition [1]. Pituitary adenomas are one of the most common intracranial neoplasms, with an estimated prevalence of approximately 17% in the general population [2]. Cross-sectional studies from various countries have reported prevalence rates ranging from 78 to 116 cases per 100,000 individuals, translating to roughly 1 case per 1,000 people [3, 4]. The incidence is higher in women during early adulthood, with a mean age of 48, while men exhibit a significantly increased incidence later in life, with a mean age of 57 [5, 6]. Furthermore, across all age groups, men tend to present with larger median tumor sizes [6]. These tumors are typically benign but often exhibit local invasiveness, leading to incomplete resection, early recurrence, lack of biochemical remission, visual impairment, and complications such as pituitary hormone dysfunction [7]. 

Recurrence rates for pituitary tumors demonstrate significant variability depending on the treatment approach. For example, non-secreting pituitary adenoma managed with surgical resection alone reported 5-year recurrence rates ranging from 15% to 55%, while 10-year recurrence rates range from 44% to 78%. The addition of adjuvant radiotherapy has consistently been shown in the literature to significantly reduce recurrence rates, highlighting its efficacy in long-term disease control [8]. 

Morbidity and mortality in pituitary adenoma are influenced by tumor type, hormonal activity, and treatment outcomes. Non-functioning tumors primarily cause mass effects, with post-treatment recurrence rates up to 78% at 10 years without radiotherapy. Historically, they have been associated with increased sick leave and disability retirement when compared to the general population [9]. Functioning tumors are associated with increased morbidity and mortality, often due to cardiovascular or metabolic complications. Aggressive subtypes exhibit poor outcomes despite multimodal treatment, underscoring the importance of early diagnosis and tailored therapy to mitigate disease burden and treatment-related sequelae​ [10, 11]. 

Given the high prevalence and rate of invasion/recurrence in young patients, early diagnosis and appropriate management of invasive pituitary neuroendocrine tumors are crucial to prevent long-term morbidity. The problem is that there are no accurate diagnostic methods for determining future malignancy risk. All aggressive pituitary adenoma are invasive by definition, so appropriately quantifying invasiveness could help develop appropriate long-term prognostic biomarkers. Although existing classification systems offer valuable guidance for managing pituitary tumors, they do not predict whether a tumor has invasive/malignant *potential*; they only assess its current invasive status. Timely intervention is critical. Studies have shown that analysis of biomarkers in the post-operative pathologic setting can predict post-operative complete remission or tumor progression [12]. The goals of this study were to perform a comprehensive review of the current literature on the local invasiveness of pituitary adenomas, specifically define biological underpinning of local invasion, and finally to evaluate the role of specific biomarkers contributing to biologically invasive phenotypes for candidacy in radiotracer studies. The specific aim of this study is to review the current literature and explore potential radiologic biomarkers that may help better predict which patients could benefit from more aggressive early treatment.

## Methods

We performed a comprehensive literature review focusing on five groupings of biomarkers potentially linked to local tissue invasion seen in pituitary adenoma. Relevant papers were identified through a systematic search of the Embase and PubMed databases. The searches, conducted in October 2025 with no date restrictions applied, targeted studies with the following terms listed in Table 1.

**Table 1:** Search terms for each biomarker reviewed, name of papers retrieved, and papers included and excluded from this study. Terms were searched for in the titles or abstracts of articles in embase and pubmed databases

Biomarker	Marker-specific search terms	Shared search term	Papers Reviewed	Papers included/excluded
**MMPs**	MMP or metalloproteinase	Invasive or invasiveness Pituitary adenoma, pituitary adenoma, pituitary mass, or pituitary tumor	81	6/75
**uPA**	uPA or urokinase-type plasminogen activator		2	1/1
**MYO5A**	MYO5A, Myosin 5a, Myosin Va (including lowercase variations)		1	1/0
**VEGF**	VEGF or vascular endothelial growth factor		76	10/66
**Survivin**	Survivin (including the lowercase variation)		11	1/10

For each biomarker, we summarize the evidence supporting its association with invasiveness and discuss the proposed mechanisms underlying its role in tumor progression. The cited *database searching tool* was used to search over all available publication years and retrieve unique papers [13]. Studies were then screened for inclusion based on their analysis of the correlation or association between the biomarker expression and pituitary adenoma’ invasiveness. Meta-analyses were prioritized for review, and any individual studies included within these meta-analyses were excluded from further analysis to prevent redundancy. The evaluation of the availability and applicability of radiolabeling techniques for their detection concludes this review.

Additionally, we applied QUADAS-2 only to primary diagnostic accuracy studies (e.g., radiolabeled imaging or other index tests compared to a reference standard for invasiveness). Meta-analyses and molecular association studies (IHC/RT-PCR/ELISA without a diagnostic accuracy framework) were excluded from QUADAS-2 and summarized.

## Results

### Local invasion as a distinct biological process

#### Prevalence of invasion

**Fig. 1:**
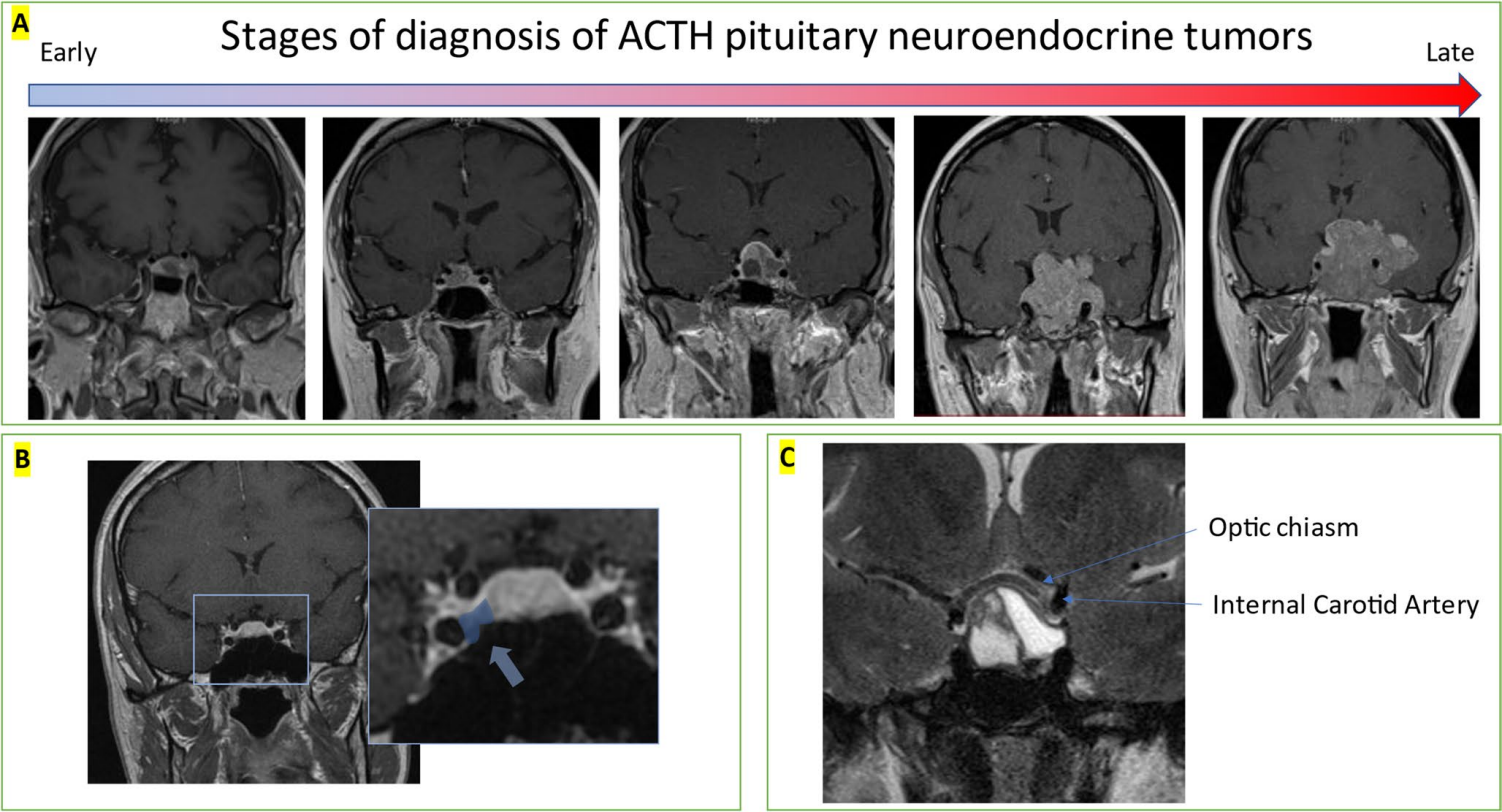
Stages of diagnosis. Panel A shows a spectrum of early to late diagnosis of silent ACTH (corticotroph) pituitary neuroendocrine tumors. Left showing early diagnosis of invasion and right showing later diagnosis of highly invasive tumor with optic nerve compression, vision loss, and brain invasion. Panel B shows an incidentally found right sided lesion with early cavernous sinus invasion. Panel C shows high resolution T2 MRI which is able to identify critical parasellar structures including optic chiasm and internal carotid artery. Despite extension to surrounding areas this pituitary adenoma is not invasive

Invasion of surrounding structures occurs in 35% of pituitary adenomas, despite the fact that these tumors are considered histologically benign [14, 15]. This invasion can occur superiorly through the diaphragma sellae, inferiorly into the basal dura towards the sphenoid sinus, or laterally into the cavernous sinuses [15]. Fig. 1 shows a progression of ACTH tumors from early to late diagnosis. Determining whether a particular tumor will become invasive or distinguishing it from a malignant pituitary tumor can be challenging. Metastatic pituitary adenomas, which account for only 0.13% of pituitary tumors, are associated with a markedly higher mortality rate [16, 17]. 

#### Classification of invasion

**Fig. 2:**
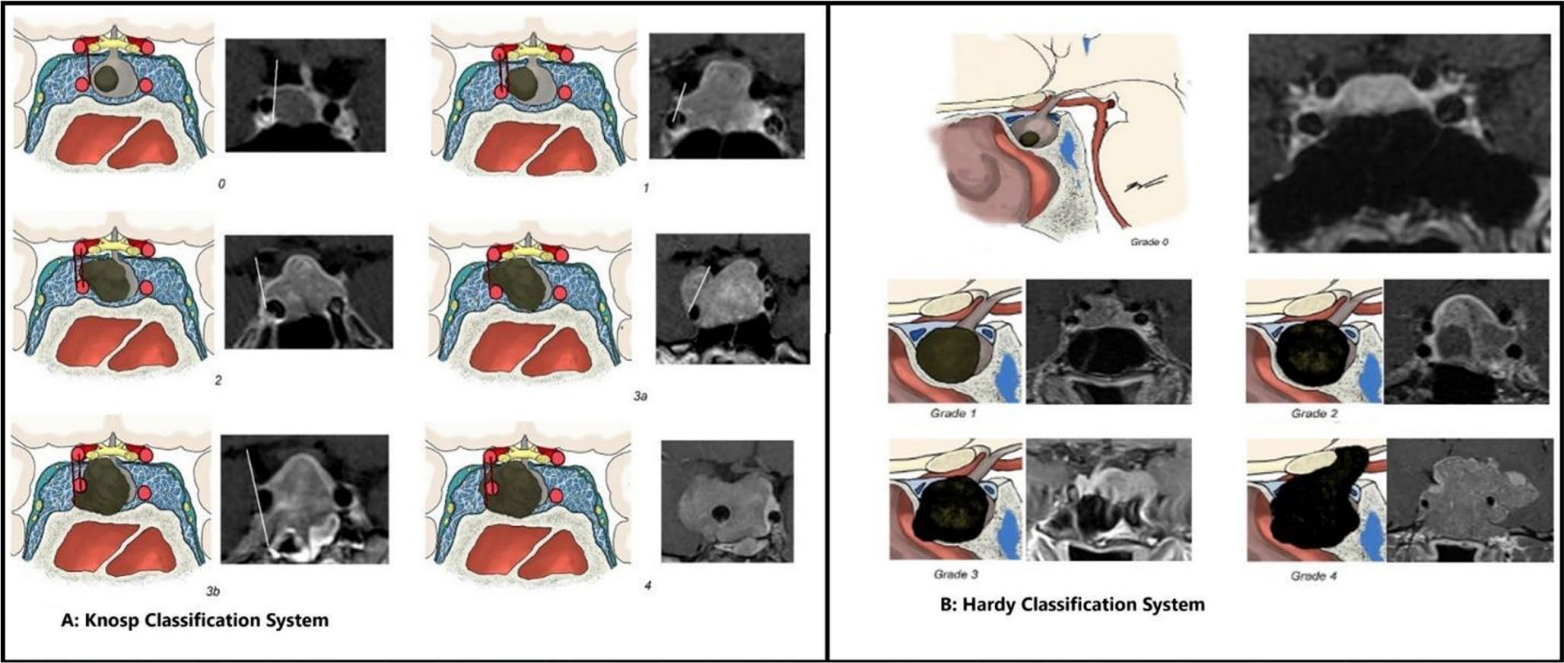
A visualization of the stages of the Knosp and Hardy classifications and accompanying MRI images

High rates of incidentally found pituitary adenoma, as described above, necessitate radiographic classification of invasiveness to predict long-term clinical outcomes as well as biological aggression. A few commonly used imaging-based grading scales for pituitary tumors are the Knosp, Hardy, and Trouillas classifications. The Knosp classification system consists of grades 0–4 (including 3a and 3b), with higher grades indicating a greater likelihood of cavernous sinus involvement and lower rates of gross total resection [18]. The scale has shown strong overall interrater reliability, but this reliability is weaker for middle grades [19]. Dichotomizing the scale into low (0–2) and high (3–4) grades or further into high (3b-4), medium (2-3a), and low (0–1) improves reliability and clinical utility [19, 20]. However, intermediate grades 2 and 3a continue to present an uncertain prognosis with regard to invasion, as approximately 30–44% of tumors in these categories are found to infiltrate the cavernous sinus [20]. Studies additionally suggest that the Knosp scale alone may not fully predict tumor behavior and resectability [21]. A visualization of this grading system is shown in Fig. 2, Panel A [22]. The grading is assigned based on coronal images and the lateral spread of the tumor based on tangents drawn from the carotid arteries, as well as the degree of encapsulation of the carotid arteries by the tumor [22]. 

The Hardy-Wilson classification system evaluates the tumor based on the extent of sellar invasion and suprasellar extension, categorizing tumors into grades 1 through 4. It has been shown to be outperformed by the Knosp scale in predicting surgical outcomes. A visualization of the Hardy-Wilson grading scale is outlined in Fig. 2, Panel B. The classification is based on the invasion of pituitary adenoma into the sella turcica and into adjacent structures [22]. 

The Trouillas classification system leverages both radiological characteristics as well as pathological characteristics to classify pituitary adenoma into one of 5 separate classifications. Grade 1a is reserved for non-invasive, non-proliferative tumors, while grade 1b is defined as non-invasive yet proliferative tumors. Grade 2a tumors are invasive, but not proliferative, while 2b tumors are both invasive and proliferative. Grade 3 tumors are metastatic tumors in which there are cerebrospinal or systemic metastases. Invasion is determined by histological or radiologic evidence of cavernous or sphenoid sinus invasion. Proliferation is defined as two out of three of the following criteria: Ki-67 >1%, mitoses with *n* >2 in 10x high-power field (HPF), or P53 positive in >10 nuclei in 10x HPF [12]. 

#### Definition of invasive pituitary adenomas

The behavior of pituitary tumors spans a continuum from invasion to aggressiveness to carcinoma. Invasion refers to the infiltration, penetration, incorporation, or destruction of adjacent tissues, and is observed in up to 35% of pituitary adenomas (approximately 350 per 1,000 cases). The European Society of Endocrinology defines aggressive pituitary tumors as those that are radiologically invasive and demonstrate unusually rapid or clinically significant growth while remaining refractory to standard therapies, including surgery, endocrine therapy, and radiation. These aggressive adenomas represent roughly 2% of macroadenomas (20 per 1,000 cases). At the most malignant end of the spectrum lies pituitary carcinoma, characterized by metastatic spread within or beyond the central nervous system. Although rare—comprising up to 0.4% of surgically resected tumors and with a population prevalence near 1 per 1,000,000—pituitary carcinomas represent the terminal manifestation of tumor progression. This tripartite classification underscores a progression from localized tissue invasion to systemic dissemination, reflecting the biological heterogeneity and clinical complexity of corticotroph and other pituitary neuroendocrine tumors [10, 23–25]. 

### Matrix metalloproteinases

#### MMP promotes tissue invasion through the degradation of type IV collagen and promotes angiogenesis

Matrix metalloproteinases (MMPs) are thought to promote pituitary tumor invasion via multiple mechanisms. The primary proposed mechanism involves the degradation of the extracellular matrix (ECM), compromising its structural integrity, and thus allowing for invasion [26]. The dura mater—largely composed of type IV collagen—is particularly susceptible, as MMP-9 is a type IV collagenase [26]. Additionally, multiple MMPs have been positively associated with angiogenesis, further contributing to tumor progression [27]. 

**Fig. 3:**
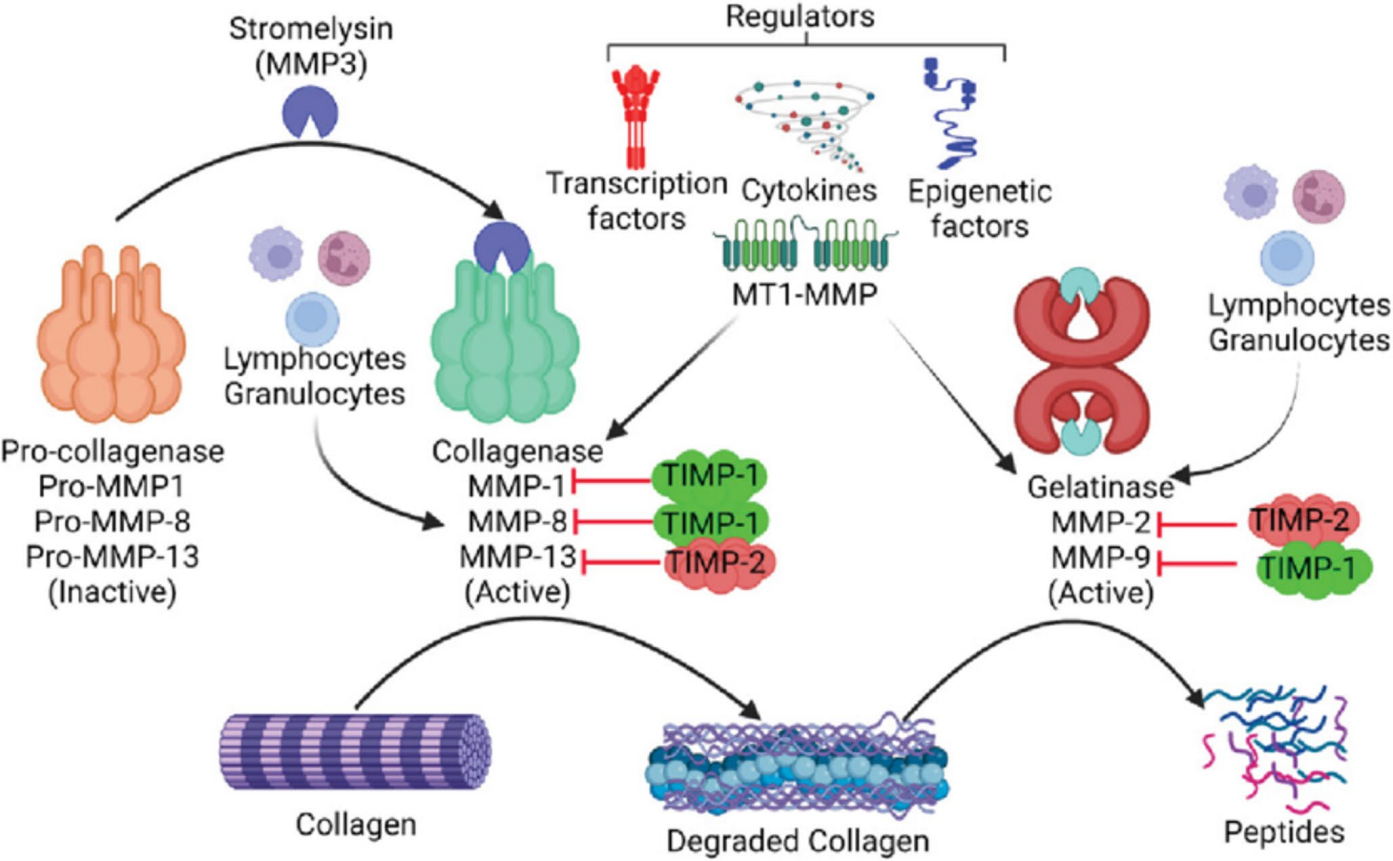
Regulation of collagen degradation by MMPs

Given the established association between elevated MMP expression and invasive pituitary adenoma, one study hypothesized that this increased expression could be linked to heightened DNA methylation of promoters of tissue inhibitors of matrix metalloproteinases (TIMPs), a phenomenon they also observed in invasive pituitary tumors [26]. This is demonstrated in Fig. 3 [28]. The combination of relatively elevated MMP levels as well as decreased TIMP levels is proposed to lead to increased degradation of collagen of the ECM, allowing for tumor invasion.

Metalloproteinases (MMPs) are some of the more well-studied potential markers for tumor invasion. Multiple studies have demonstrated significantly higher expression of MMP-9 and/or MMP-2, among others, in invasive pituitary adenoma compared to non-invasive tumors. The results of a literature review are described in detail in Table 2. All studies showed a statistically significant association between MMP-9 and invasiveness, with the exception of one, as determined by multiple methods. The included studies covered 2,773 cases of pituitary tumors. MMP-2 was discussed in a single meta-analysis, which also found that there was a significant association between MMP-2 presence in tumors and invasiveness, as well as a study that found no statistically significant association [27, 29–33]. In conclusion, MMPs are clearly activated in pituitary neuroendocrine tumor invasion; however, the exact mechanism remains unclear.

**Table 2:** Description of studies that met the criteria to be analyzed for MMPs 9 and 2. Year column contains information on data collection and research publication. Gong J et al. is described in detail in Table 2

Author	Year	Location	Subject	Patient Population	Methods	Results
**The B Friere et al.**	2014 to 2015	Hospital das Clinicas HCFMUSP	Evaluate association of MMP-2 and MMP-9 expression with CSI	74 patients 37 male, 37 female	Immunohistochemistry staining of tumor samples for evaluation of the aforementioned markers	No statistically significant difference in expression of MMP-2 or MMP-9 between invasive and noninvasive groups.
**Liu HYet al.**	1996 and 2015/2016	Multinational	The association between MMP-9 and MMP-2 expression and pituitary adenoma invasiveness	24 Articles 1,320 patients	Meta-analysis of case-control studies Invasiveness was defined by a modified Hardy classification grade of III-IV, Knosp grade III-IV, surgeon-confirmed penetration into adjacent structures, or damage to surrounding tissues Identification using either RT-PCR or IHC	MMP-9 expression in invasive tumors was examined by 21 studies, yielding an odds ratio of 5.48 with a 95% confidence interval of 2.61–11.5 (*P* < 0.00001). 11 studies: association of MMP-2 expression with invasiveness, reporting an odds ratio of 3.58 and a 95% confidence interval of 1.63–7.87 (*P* = 0.001). An association between the presence of MMP-9 and MMP-2 and the invasiveness of pituitary adenoma has been shown.
**Sun Bet al.**	2020	UK, Germany, Turkey and China	The association of MMP-9 and pituitary adenoma invasiveness	22 articles 1,153 Patients: 997 Chinese, 156 in UK, Germany, and Turkey Median age 42 years	Meta-analysis Positive staining of the nucleus or cytoplasm in pituitary tumor tissue Various methods were used by studies to determine invasiveness	514 of the 631 patients with reported invasive tumors demonstrated positive MMP-9 with an odds ratio of 4.75 and a 95% confidence interval (CI) of 3.53 to 6.39 (*p* = 0.000). Some publication bias was identified in the meta-analysis, as indicated by Begg’s and Egger’s tests
**Guo Het al.**	2010 and 2012/2019	Harrison Int’l Peace Hospital	The association of MMP-9 and invasiveness of pituitary tumors	108 patients 61 men and 47 women Ranging 25–57 years	Stratification into invasive (*n* = 58) and non-invasive (*n* = 50) groups based on the Hardy-Wilson and Knosp classification systems IHC and western blotting for protein-level analysis and RT-PCR for gene-level evaluation	Significant elevation in MMP-9 expression in invasive pituitary tumors compared to non-invasive ones, at both protein and gene levels. IHC: MMP-9 positivity rate of 86.21% in invasive tumors, compared to 50% in non-invasive tumors. RT-PCR: approximately double the mRNA expression of MMP-9 in invasive tumors relative to non-invasive tumors. Validation by western blotting and serum ELISA, with similar results.
**Gong J et al.**	November 2005 to October 2006/2007	University of Virginia Medical Center	The association between MMP-9 expression and tumor invasiveness	73 patients 37 men, 36 women Mean age: 52 years (range 11–79)	MMP-9 expression was analyzed using quantitative RT-PCR, IHC staining, and gelatin zymography, with verification through western blot analysis. Invasion is determined by both pathology and gross observation of invasion.	Table 3 **provides a summary of this study’s results**. Significant increase in MMP-9 expression in invasive pituitary tumors compared to non-invasive tumors, in functioning and non-functioning tumors. Confirmed through RT-PCR analysis, and gelatin zymography, then validated by IHC staining
**Pan L et al.**	2005	The people’s Hospital of Dongguan	The association between MMP-9 expression and invasiveness	45 patients 25 male, 19 female	Biomarker expression was analyzed using IHC staining. Scored based on staining intensity (0–3) by percent of cells involved: 0 = 0%, 1 < 25%, 2 between 26 & 50% 3 > 50%. Invasiveness was determined intraoperatively by the absence of an intact medial wall of the cavernous sinus	The study identified statistically significant associations between pituitary tumor invasiveness and the expression of VEGF (*P* < 0.001) and MMP-9 (*P* < 0.001)

**Table 3:** The results of Gong J et al, analyzed through various methods, pertaining to all pituitary adenoma, and both functional and non-functional types

Type of pituitary adenoma	Technique	Non-Invasive Expression*	Invasive Expression*	P-Value
All	RT-PCR	0.58 ± 0.09 fold	2.32 ± 0.38 fold	P < 0.01
Gelatin Zymography	1.03 ± 0.20 ng/ml	2.37 ± 0.33 ng/ml	P < 0.01	
Functional Tumors	RT-PCR	0.52 ± 0.12 fold	2.55 ± 0.76 fold	P < 0.01
Gelatin Zymography	1.41 ± 0.19 ng/ml	3.23 ± 0.65 ng/ml	P < 0.05	
Non-Functional Tumors	RT-PCR	0.61 ± 0.13 fold	2.21 ± 0.44 fold	P < 0.01
Gelatin Zymography	0.77 ± 0.21 ng/ml	1.94 ± 0.34 ng/ml	P < 0.01	

* For RT-PCR, results were evaluated by calculating the fold increase over control, expressed as the ratio of the relative quantities of treated and experimental samples with means calculated via ANOVA. Gelatin zymography results were assessed by comparing activity through densitometric analysis and semi-quantitative measures

#### Summary of radiolabels for MMP

In a 2001 study, researchers outlined the methodology for synthesizing the radiolabeled compound ^111^In−DTPA − N-TIMP-2, designed to bind and inhibit MMP-3. This radiolabel was created by conjugating the MMP inhibitor TIMP-2 with diethylenetriamine pentaacetic acid (DTPA) and then radiolabeling it with indium-111. The resulting compound demonstrated serum stability over 48 h, with approximately 5% of the radiolabel dissociating during this time. Furthermore, the radiolabel retained its inhibitory activity, was pyrogen-free, and met sterility standards [34]. Given the established association between pituitary tumors and elevated levels of MMP-9—and, to a lesser extent, MMP-2—there is potential for further research to develop radiolabels targeting these specific molecules.

A 2014 study reported the development of activatable cell-penetrating probes designed to assess MMP-2 and MMP-9 activity, though in the distinct context of post-myocardial infarction cardiac remodeling in murine models. These probes were engineered to be cell-permeable and activatable upon interaction with MMPs, enabling real-time imaging of enzymatic activity [35]. While not applied to pituitary adenoma, the methodology highlights a potential avenue for utilizing similar probes to evaluate MMP activity in the context of invasive pituitary tumors, offering new insights into tumor behavior and potential diagnostic strategies.

The methodological quality of these radiolabeled imaging studies was evaluated using the QUADAS-2 tool, assessing risk of bias across patient selection, index test, reference standard, and flow/timing domains. The results are summarized in Table 5. Overall, the 2001 study showed a high risk of bias due to a lack of a true diagnostic reference standard, whereas the 2014 ACPP study demonstrated a low risk of bias within its preclinical design but limited clinical applicability.

### Urokinase-type plasminogen activator (uPA)

#### uPAR is an upstream receptor that promotes invasiveness through activation of MMPs

**Fig. 4:**
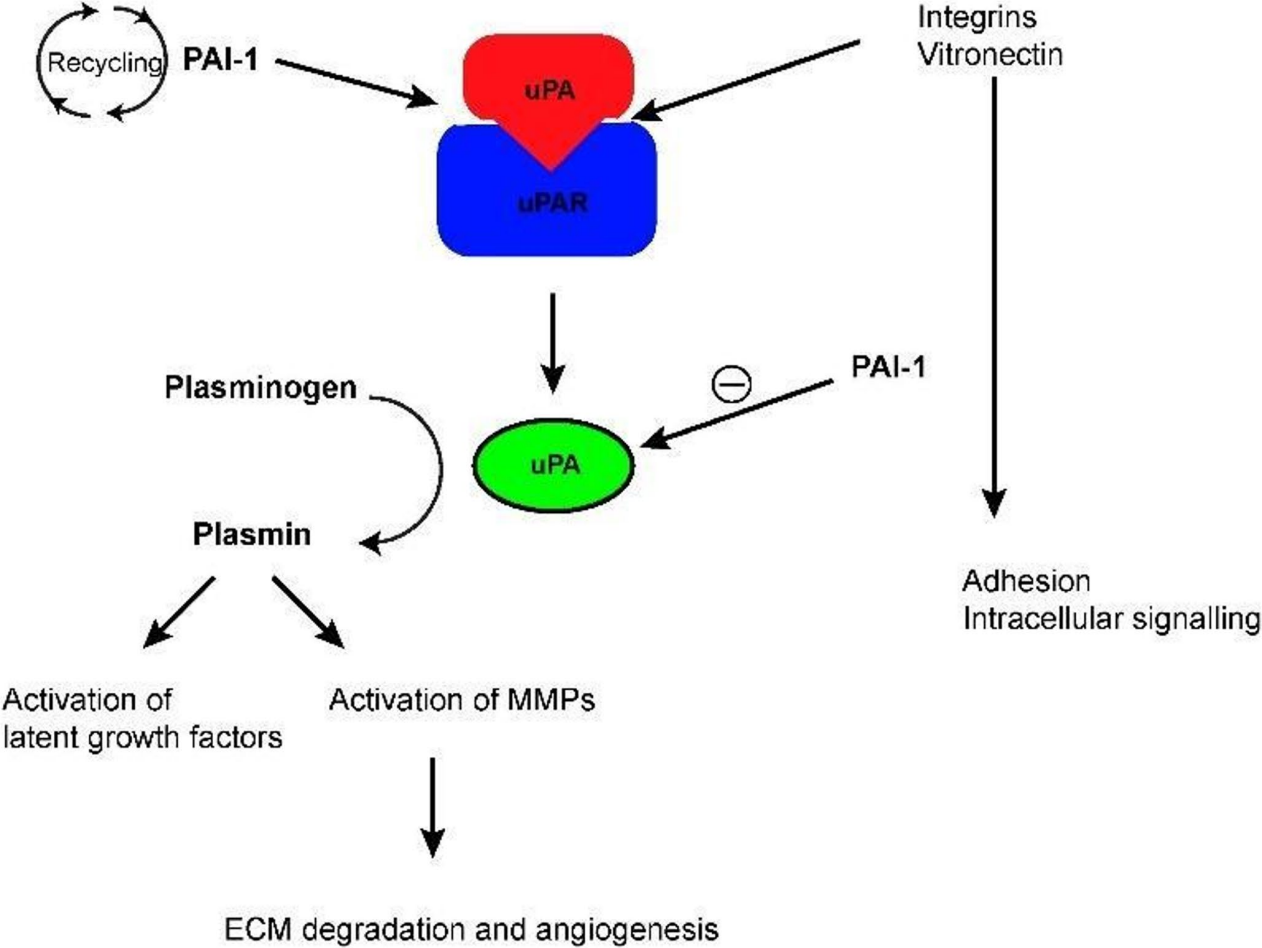
Regulation of uPA and activation of plasmin

The uPA system is proposed to contribute to multiple processes involved in carcinogenesis, tumor invasion, and metastasis. Its primary function is the conversion of plasminogen to plasmin, which facilitates the proteolytic degradation of the basement membrane and extracellular matrix through the activation of MMPs, thus allowing invasion and metastasis [36]. Moreover, the uPA system has been shown to promote epithelial-mesenchymal transition, activate mitogenic signaling pathways, inhibit apoptosis, and enhance cell migration, invasion, and metastasis [36]. Fig. 4 includes a visual representation of the uPA system [37]. 

The uPA system has been widely reported in the literature to be associated with tumor invasiveness and metastatic potential across various malignancies, with significant prognostic implications [38]. 

#### uPA overexpression is weakly associated with pituitary tumor invasiveness

A 2003 blinded study examined the expression of uPA and uPA receptor (uPAR) concerning the invasiveness of both functional and nonfunctional pituitary tumors. Samples were collected from 84 patients at the University Medical Center Hamburg-Eppendorf during transsphenoidal surgery. The patients had a mean age of 47 years, ranging from 17 to 79 years. Tissue expression of uPA was assessed using immunohistochemical staining, and four independent, blinded observers rated the expression on the following scale: “distinct” for >50% of positively stained cells, “moderate” for < 50%, “slightly positive” for small clusters or weak diffuse reactions, and “negative.“ [39].

uPA and uPAR were expressed in 89% and 90% of pituitary tumors, respectively. Staining was positive in 90% and 92% of invasive tissues for uPA and uPAR, respectively, and in 88% of non-invasive tissues for both markers. Invasiveness was determined via preoperative MRI and verified by intraoperative inspection. Expression levels of uPA and uPAR did not correlate with invasiveness in functional pituitary tumors, though uPA showed a stronger tendency toward overexpression in invasive compared to non-invasive non-functional tumors (Chi-square *P* = 0.053). uPAR did not show any association with invasiveness [39]. 

#### Summary of radiolabels for uPA and uPAR

Several successful attempts have been made to conjugate DOTA (1,4,7,10-tetraazacyclododecane-1,4,7,10-tetraacetic acid) and NODAGA (1-(1-carboxy-3-carboxy-propyl)−4,7-(carboxy-methyl)−1,4,7-triazacyclononane) with various radionuclides to target uPAR in mouse models [40]. However, these studies have reported confounding levels of non-specific baseline tracer accumulation in both liver and tumor tissue, particularly with ^64^Cu-labeled markers [40]. 

The methodological quality of this imaging study was assessed using the QUADAS-2 framework (Table 5). Risk of bias was low for the index test—radiolabel synthesis, validation, and imaging procedures were well described and blinded to biodistribution data—but high for patient selection and reference standard domains, as all experiments were performed in murine xenografts without histologic confirmation of uPAR expression. Flow and timing were adequate, with consistent imaging and biodistribution intervals.

### Myosin 5a (MYO5A)

#### MYO5A expression is upregulated when bound to Snail and Akt and associated with invasion through the epithelial-mesenchymal transition (EMT) pathway

MYO5A is an unconventional myosin protein that is upregulated by EMT markers such as Snail and Akt2. It is additionally proposed that Snail could be a transcriptional activator of MYO5A. EMTs promote the loss of epithelial characteristics of epithelial cells and induce the gain of mesenchymal characteristics, which allows for loose adherence and increased motility. These EMTs, and MYO5A by extension, are associated with increased invasiveness and metastasis [41, 42]. 

Increased expression of MYO5A has been observed in several metastatic cell lines, including metastatic colorectal, lung, breast, and prostate cancers, as well as in invasive and metastatic esophageal squamous cell carcinomas [41, 42]. 

#### Myosin 5a gene expression is elevated in invasive pituitary tumors

A 2010 study analyzed a sample of 40 non-functional pituitary adenoma resected via transsphenoidal surgery at Bicêtre Hospital in France. The study assessed gene expression and protein levels using RT-PCR and immunohistochemical (IHC) staining, respectively, including the MYO5A gene and protein. Tumor invasiveness was classified based on preoperative and intraoperative MRI findings, employing the modified Hardy grading scale. Grade III and IV tumors were categorized as invasive. Of the tumors evaluated, 22 were classified as invasive, while the remaining 18 were not [43]. The study demonstrated that MYO5A gene expression was significantly elevated in invasive tumors compared to noninvasive tumors, with a corresponding increase observed at the protein level [43]. 

#### Summary of radiolabels

As of the time of this paper’s writing, research on radiolabels targeting MYO5A remains limited.

### Vascular endothelial growth factor (VEGF)

#### Hypoxia-induced VEGF upregulation promotes angiogenesis

Tumor hypoxia is thought to upregulate VEGF expression, promoting its release within the tumor microenvironment [44]. VEGF and its receptor are known to trigger angiogenesis and are correlated with invasiveness and metastaticity, but the mechanisms by which VEGF/VEGFR1 may promote these tumor characteristics remain unknown [45]. 

VEGF and VEGF receptor 1 (VEGFR1) expression has been documented to be associated with invasion and metastasis for various types of cancers, including head and neck squamous cell cancer and bladder cancer, among others [44, 46, 47]. 

Nine studies are outlined in Table 4 below with their respective results. Six of these nine studies demonstrated positive results for increased VEGF expression in invasive pituitary tumors, while the remaining 3 were not able to identify any statistically significant difference between the two groups. These studies included 450 patients in total [48–56]. The variability in findings regarding the association of VEGF with invasion suggests that it may not be the most sensitive biomarker for assessing invasive potential.

**Table 4:** Descriptions of studies that met the criteria to be analyzed for VEGF and VEGFR. Year column contains information on data collection and research publication. Pan L et al. is described in detail in Table 2. *CSI = cavernous sinus invasion

**Sato**,** M et al.**	2011–2017	Keio University School of Medicine	VEGF & VEGFR1 expressions in nonfunctional pituitary adenoma	27 patients	IHC staining for VEGF and VEGFR1 Invasiveness determined by CSI* on Gd-enhanced T1 MRI	Statistically significant increases in VEGF and VEGFR1 expression in tumors that invaded the cavernous sinus (*P* = 0.033 and *P* = 0.04, respectively)
**Baldys-Waligorska A et al.**	1997–2008	Jagiellonian University Medical College	VEGF expression levels in a cohort of patients diagnosed with somatotropinomas	31 patients	Retrospective analysis of clinical, histopathological, and IHC records Invasiveness via Knosp grading system & surgeon descriptions.	The analysis did not reveal statistically significant differences in VEGF expression between invasive and non-invasive tumors
**He W et al.**	2017	2nd Affiliated Hospital of Nanchang University	The association between VEGF-A expression and the invasiveness of pituitary tumors	76 patients 32 males and 44 females	RT-PCR and western blot analyses to Invasivness determined by Hardy-Wilson and Knosp grading system.	VEGF-A expression, at both the mRNA and protein levels, was significantly elevated in the invasive pituitary tumor group compared to the non-invasive group
**Yilmaz M et al.**	2003–2008	Kocaeli University Hospital	VEGF expression as a parameter of cavernous sinus invasion	28 patients	IHC to determine expression. Invasiveness defined as Knosp of 4 or 1–3 with: compression of 3 + venous compartments or > 45% encasement of ICA.	Statistical analysis using the Chi-Square test revealed a significant positive association between VEGF expression and cavernous sinus invasion (*p* = 0.03)
**Sánchez-Ortiga Ret al.**	1995–2008	Hospital General Universitario de Alicante	VEGF levels of invasive pituitary tumors	46 pituitary tumor samples. 39 with extrasellar growth.	RT-PCR Invasiveness determined by tumor affecting surrounding structures on presurgical MRI imaging.	Significantly higher normalized copy numbers in tumors with extrasellar growth compared to those without (0.566 vs. 0.200; *p* = 0.008). The study further proposed a normalized copy number threshold of 0.222, which was associated with a 27.5-fold increased risk of extrasellar growth (*p* = 0.002)
**Tanase C et al.**	2013	Victor Babes National Institute of Pathology	Association between invasiveness & serum VEGF levels in pituitary tumors	66 samples 43 invasive & 23 non-invasive	xMAP and ELISA	The study identified significant differences in serum VEGF levels between invasive and non-invasive pituitary tumor
**Yarman Set al.**	2010	Istanbul University	Expression of VEGF in invasive and non-invasive PAs	47 GH-secreting pituitary adenoma 21 invasive & 26 non-invasive	IHC scoring system adding: ● Staining Intesity: 0 = negative, 1 = weak, 2 = intermediate, 3 = strong ● % cells staining: 0 = 0%, 1 < 25%, 2 < 50%, 3 ≥ 50%	A significant positive association between VEGF positivity and tumor invasiveness (*p* = 0.016)
**Borg SA et al.**	2005	University of Sheffield Medical School	The relationship between VEGF protein expression and tumor invasiveness	59 pituitary tumor samples	ELISA to determine expression Invasiveness determined by Hardy classification system of 3 & 4 on pre-operative MRI scans	Although invasive tumors demonstrated higher mean VEGF secretion levels compared to non-invasive tumors (2054 ± 1608 pg/mL vs. 608 ± 143 pg/mL), this difference was not statistically significant (*p* = 0.867)
**Iuchi T et al.**	2000	Chiba Cancer Centre	association between VEGF expression and invasiveness	25 growth hormone-secreting pituitary tumors	IHC Invasiveness determined by Knosp grades 3 and 4 on preoperative imaging.	The study found no statistically significant difference in VEGF expression between invasive and non-invasive growth hormone-secreting pituitary tumors
**Pan L et al.**	2005	See the last entry in Table 2 for more details				

#### Radiolabels for vascular endothelial growth factor and receptor

Multiple studies have explored the development of radiolabels targeting VEGFR1. These include ^99^mTc-labeled VEGF165 for SPECT imaging [57], 177Lu-labeled anti-VEGFR1 antibodies for radioimmunotherapy [58], and ^89^Zr-labeled single-chain VEGF mutants for selective PET imaging of VEGFR-1 and VEGFR-2 [59]. ^64^Cu-labeled VEGF121 has demonstrated utility for in vivo visualization of VEGFR expression [60], while ^68^Ga-labeled peptides and other small-molecule VEGFR inhibitors targeting VEGFRs show promise for PET imaging [61, 62]. Radiolabeled bevacizumab, an anti-VEGF antibody, has additionally been developed for PET/SPECT imaging of VEGF in tumors using ^89^Zr and ^111^In [63]. Methodological quality was assessed using QUADAS-2 in Table 5.

### Survivin

#### Survivin promotes tumor invasion by preventing cell death and promoting cell motility

Survivin is thought to inhibit cell apoptosis and thereby contribute to tumor cell proliferation. Its role in promoting invasion is believed to involve multiple mechanisms. Research has shown that survivin enhances oxidative phosphorylation and mitochondrial repositioning, facilitating cellular respiration to generate the energy required for tumor cell migration and invasion [64]. Furthermore, another study demonstrated that the formation of a survivin-XIAP complex within tumor tissue promotes cell motility, contributing to invasion and metastasis [65].

Increased expression of survivin has been found to be increased in a variety of malignant tumors, including lung cancer, breast cancer, and gliomas [66]. 

#### Survivin expression is associated with pituitary tumor invasion

A 2017 meta-analysis examined 9 studies conducted across Asia and Europe, involving a total of 489 patients, 266 of whom had invasive pituitary tumors. Survivin expression was associated with tumor invasiveness in these studies, with 8 using IHC staining and 1 using RT-PCR. Positive survivin expression was defined as staining in the nucleus or cytoplasm. Of the 9 studies, 7 showed statistically significant associations between survivin expression and increased invasiveness. This resulted in an odds ratio of 6.23, with a 95% confidence interval of 3.97 to 9.76 (*P* < 0.001) [66]. 

#### Radiolabels for survivin

Several radiolabeled compounds targeting survivin have been developed for tumor imaging, including radioiodinated 4,6-diaryl-3-cyano-2-pyridinone derivatives [67] and 3-phenethyl-2-indolinone derivatives [68]. These compounds demonstrated specific binding to survivin in vitro murine models and showed moderate tumor uptake in vivo [68]. While these probes established the feasibility of survivin-targeted molecular imaging, their preclinical design and limited histopathologic validation result in a high risk of methodological bias as summarized by QUADAS-2. The QUADAS-2 evaluation is shown in Table 5.

**Table 5:** QUADAS-2 Bias assessment of radiolabels for biomarkers

Biomarker	Study	Year	Index Test	Reference Standard	Patient Selection	Index Test	Reference Standard	Flow/Timing
**MMP**	Giersing et al.	2001	¹¹¹In-DTPA-N-TIMP-2	Ex vivo MMP-2 binding/biodistribution	High	Low	High	Low
	van Duijnhoven et al.	2014	¹⁷⁷Lu/¹²⁵I-ACPP, Alb-ACPP (uPAR PET)	Gelatin zymography (MMP-2/9 activity)	Low	Low	Low	Low
**uPA**	Ploug et al.	2013	^64^Cu-DOTA-AE105	Ex vivo biodistribution and blocking control	High	Low	High	Low
**Survivin**	Fuchigami et al.	2016	¹²⁵I-IDCP (SPECT)	In-vitro binding + biodistribution	High	Low	High	Low
**VEGF**	Lee et al.	2009	¹⁷⁷Lu-anti-VEGFR1 mAb (radioimmunotherapy)	Tumor growth and VEGFR1 expression by IHC	High	Low	High	Low
	Galli et al.	2017	⁹⁹ᵐTc-VEGF₁₆₅ (SPECT)	Biodistribution and blocking control	Low	Low	Unclear	Low
	Meyer et al.	2016	⁸⁹Zr-scVEGF mutants (PET)	Ex vivo biodistribution + VEGFR1/2 IHC	Low	Low	Unclear	Low
	Cai et al.	2006	⁶⁴Cu-DOTA-VEGF₁₂₁ (PET)	Biodistribution and blocking control	Low	Low	Unclear	Low
	Nagengast et al.	2007	⁸⁹Zr-bevacizumab (PET)	IHC VEGF-A levels + therapy response	Unclear	Low	Unclear	Low

## Discussion

The findings from our review underscore the critical role of biological invasion of pituitary adenoma management. Elevated matrix metalloproteinases (MMP) expression was found to correlate with increased tumor invasiveness, likely through extracellular matrix degradation and angiogenesis promotion as seen in other locally invasive tumors [26, 27, 29–33, 69]. The observation that DNA methylation of TIMP promoters may contribute to unchecked MMP activity further supports the hypothesis that the MMP-TIMP balance plays a key role in tumor progression [26]. Despite the compelling evidence linking MMPs to invasion, the exact regulatory mechanisms remain unclear, warranting further research into their upstream signaling pathways and interactions.

Beyond MMPs, the uPA and uPAR system has been implicated in tumor invasion through its activation of MMPs and facilitation of EMT [36]. However, direct association with pituitary tumor invasiveness remains inconclusive. The single 2003 study observing invasiveness with the presence of these biomarkers determined invasiveness via MRI and surgeon observation, rather than the gold standard of pathologic analysis [39]. Novel research observing uPA or uPAR expression with pathologic invasion may be beneficial and give more conclusive results.

Myosin 5a (MYO5A) emerges as another promising marker, with studies demonstrating its upregulation in invasive pituitary tumors [43]. MYO5A’s interaction with EMT markers such as Snail and Akt further supports its role in facilitating tumor cell motility and metastasis [41, 42]. However, research on MYO5A remains limited, highlighting the need for additional studies to validate its potential as a diagnostic or therapeutic target.

Vascular endothelial growth factor (VEGF) has been widely studied for its role in tumor angiogenesis. Although several studies demonstrate an association between VEGF upregulation and pituitary tumor invasion, findings are inconsistent, with some failing to identify a statistically significant correlation. This suggests that VEGF may not be the most reliable independent predictor of invasiveness in pituitary tumors [48–56]. 

Survivin, a well-established inhibitor of apoptosis, appears to contribute to tumor invasion by enhancing cell motility and metabolic adaptation [64]. Meta-analysis findings indicate a strong association between survivin expression and pituitary tumor invasiveness, with significant odds ratios supporting its prognostic value [66]. Given its involvement in multiple malignancies, survivin presents as a compelling target for future research into invasive pituitary tumors.

The exploration of radiolabeled compounds for imaging and therapeutic targeting of these markers represents an exciting frontier in neuroendocrine-oncology. Radiolabeled inhibitors for MMPs, uPA/uPAR, VEGF, and survivin have demonstrated feasibility, with several compounds showing promising tumor specificity and in vivo stability. However, challenges such as non-specific tracer accumulation, particularly with 64Cu-labeled uPAR markers, indicate the need for further refinement of radiolabeling strategies.

In conclusion, this review highlights the multifactorial nature of pituitary tumor invasion, driven by MMPs, the uPA system, MYO5A, VEGF, and survivin. While each marker contributes to tumor progression through distinct mechanisms, their combined effects likely orchestrate the invasive phenotype. Future research should focus on elucidating the interplay between these pathways, developing targeted imaging agents, and refining therapeutic interventions to mitigate tumor invasion and improve patient outcomes.

### Limitations

A major limitation of the research discussed is that these studies overwhelmingly establish the association of already known and observed invasiveness, as defined by their respective methods, with biomarker presence. Whether these biomarkers are elevated in tumors that have not yet become invasive but will be, has not been investigated, and would require longitudinal studies to establish sufficient evidence.

Another significant limitation is the method used to determine the invasiveness of pituitary tumors. Many studies determine invasiveness via non-pathological methods such as imaging and surgeon observations. Both methods of determining invasiveness are subject to error and may affect the accuracy of study results, and are not considered the gold standard for determining invasiveness. Thus, associations or lack thereof may be erroneous due to the potentially inaccurate classification of tumors into invasive and non-invasive subgroups.

### Summary of results and future directions

This paper reviews the evidence linking biomarkers—MMP-2, MMP-9, uPA, MYO5A, VEGF/VEGFR, and survivin—with invasive behavior in pituitary adenoma. These markers are proposed as potential risk indicators and may provide insights into the invasive potential of tumors. Overwhelmingly, published literature has reported associations between these biomarkers and invasion. Emerging evidence also highlights the feasibility of radiolabeling certain biomarkers, suggesting a promising avenue for noninvasive assessment and prediction of tumor invasiveness, particularly in incidentally discovered pituitary lesions. These findings underscore the potential for biomarker-driven strategies in managing pituitary tumors.

The identification and utilization of biomarkers such as matrix metalloproteinases (MMPs), urokinase plasminogen activator and its receptor (uPA/uPAR), myosin 5 A (MYO5A), survivin, and vascular endothelial growth factor (VEGF) have the potential to significantly enhance the prediction of pituitary tumor invasiveness. These markers have demonstrated associations with the invasive behavior of pituitary tumors across both functional and non-functional subtypes. Further investigation into the relationship between these biomarkers (and others) and tumor invasiveness is critical to building a robust dataset that could inform the development of targeted radiolabels.

Although many radiolabels targeting these biomarkers are in the early phases of development, they hold promise for refining diagnostic algorithms and improving the management and surveillance of pituitary tumors. The utility of these radiolabels as reliable predictors of future tumor aggressiveness and invasiveness will require validation through clinical studies as they become more widely available. If these radiolabels are found to be accurate and specific predictors of invasiveness, they may also be used for the development of therapeutic interventions targeting abnormal tissue.

During the course of this review, several other biomarkers potentially associated with the invasiveness of pituitary adenoma have been identified. These included MMP-1, MMP-14, PTTG, reticulin, laminins, type IV collagen, EHZ2, estrogen receptor, HIF1, FBGF-2, LAMA2, DDR1, CLIC2, TOPO 2 A, and others. While this analysis focuses on five groups of selected biomarkers with therapeutic potential, further exploration of additional biomarkers is strongly recommended to enhance our understanding of tumor behavior.

Moreover, a logical extension of this discussion involves the investigation of biomarkers specific to metastatic pituitary carcinoma, however, given the rarity of this condition, the available data is expected to be limited. Beyond the application to pituitary carcinoma, the investigation of biomarkers of local invasion for all solid tumors would be useful, especially for neoplasms involving the central nervous system where local invasion is usually associated with increased morbidity and limited treatment options.

## Data Availability

No datasets were generated or analysed during the current study.
